# Differentially abundant proteins associated with heterosis in the primary roots of popcorn

**DOI:** 10.1371/journal.pone.0197114

**Published:** 2018-05-14

**Authors:** Mathias F. Rockenbach, Caio C. G. Corrêa, Angelo S. Heringer, Ismael L. J. Freitas, Claudete Santa-Catarina, Antônio T. do Amaral-Júnior, Vanildo Silveira

**Affiliations:** 1 Laboratório de Biotecnologia, Centro de Biociências e Biotecnologia (CBB), Universidade Estadual do Norte Fluminense Darcy Ribeiro (UENF), Av. Alberto Lamego, Campos dos Goytacazes, RJ, Brazil; 2 Unidade de Biologia Integrativa, Setor de Genômica e Proteômica, UENF, Campos dos Goytacazes, RJ, Brazil; 3 Laboratório de Melhoramento Genético Vegetal, Centro de Ciências e Tecnologias Agropecuárias (CCTA), UENF, Campos dos Goytacazes, RJ, Brazil; 4 Laboratório de Biologia Celular e Tecidual, CBB-UENF, Campos dos Goytacazes, RJ, Brazil; Shantou University Medical College, CHINA

## Abstract

Although heterosis has significantly contributed to increases in worldwide crop production, the molecular mechanisms regulating this phenomenon are still unknown. In the present study, we used a comparative proteomic approach to explore hybrid vigor via the proteome of both the popcorn L54 ♀ and P8 ♂ genotypes and the resultant UENF/UEM01 hybrid cross. To analyze the differentially abundant proteins involved in heterosis, we used the primary roots of these genotypes to analyze growth parameters and extract proteins. The results of the growth parameter analysis showed that the mid- and best-parent heterosis were positive for root length and root dry matter but negative for root fresh matter, seedling fresh matter, and protein content. The comparative proteomic analysis identified 1343 proteins in the primary roots of hybrid UENF/UEM01 and its parental lines; 220 proteins were differentially regulated in terms of protein abundance. The mass spectrometry proteomic data are available via ProteomeXchange with identifier “PXD009436”. A total of 62 regulated proteins were classified as nonadditive, of which 53.2% were classified as high parent abundance (+), 17.8% as above-high parent abundance (+ +), 16.1% as below-low parent abundance (− −), and 12.9% as low parent abundance (-). A total of 22 biological processes were associated with nonadditive proteins; processes involving translation, ribosome biogenesis, and energy-related metabolism represented 45.2% of the nonadditive proteins. Our results suggest that heterosis in the popcorn hybrid UENF/UEM01 at an early stage of plant development is associated with an up-regulation of proteins related to synthesis and energy metabolism.

## Introduction

Popcorn (*Zea mays* L.) is a high-yielding crop that is both highly popular and accepted worldwide. Breeders have widely used recurrent selection to increase the frequency of alleles coding for traits of agronomic interest without reducing the genetic variability of populations of this crop [1–3]. However, recurrent selection is often accompanied by heterosis with respect to the development of superior genotypes of several crops [4,5]. In Brazil, the use of heterosis in maize crop breeding has increased productivity approximately fourfold since the 1980s [6].

Heterosis or hybrid vigor is the superior performance of F_1_ progeny with respect to the parental lines [7]. Hybrid performance can be classified as mid-parent heterosis (MPH) or best-parent heterosis (BPH) [5,8]. MPH involves hybrid traits that display significantly better performance than the average value of the parental lines, whereas BPH involves hybrid traits that perform significantly better than those of the best parental line [5]. Although hybrids can exhibit heterotic effects for different traits simultaneously, the magnitude of heterosis can substantially vary among traits [9].

Due to the complexity of heterosis, no specific biochemical pathways that reveal a direct connection to multigenic heterosis have been established. However, several molecular approaches have been demonstrated with different trends in global metabolic regulation, protein accumulation, specific protein functions, epigenetic modification, and posttranscriptional and posttranslational modifications that also regulate heterosis in different plant organs and at different developmental stages [10–13].

As such, comparative proteomic strategies have successfully been used to identify proteins and biological processes associated with heterosis in corn [14], sunflower [15], and papaya [12]. In addition, proteomic studies have revealed an overlap of nonadditive protein accumulation between corn and rice, suggesting that organ- or tissue-specific regulatory mechanisms exist, depending on the species [16]. These studies have revealed several cellular and molecular processes associated with heterotic phenotypes in different tissues where the majority of nonadditively accumulated proteins in heterotic hybrids belong to functional classes of root development associated mainly with energy processes and production, stress responses, root development, and amino acid and protein metabolism in mitochondria [10,14,16,17].

Increased rates of root protein synthesis also play an important role in hybrids during early stages of root development for better hybrid performance. These plants can develop faster by absorbing water more efficiently, obtaining structure, and gaining independence from seed reserves sooner [14,16]. In the present work, we used a comparative proteomic approach to explore hybrid vigor by analyzing the differentially abundant proteins of both the popcorn L54 ♀ and P8 ♂ genotypes and the resultant UENF/UEM01 hybrid cross.

## Materials and methods

### Biological material and sampling

Seeds of a F_1_ popcorn hybrid (UENF/UEM01) and its parental lines (P8 ♂ and L54 ♀, “Beija-Flor” genealogy) were obtained from the Germplasm Bank of Popcorn belonging to the experimental unit in Colégio Antônio Sarlo (21°72'S/41°34'W) from the Universidade Estadual do Norte Fluminense Darcy Ribeiro (UENF), Rio de Janeiro (RJ), Brazil.

The seeds of all genotypes were selected in sieves for sizes greater than five and smaller than six millimeters in diameter. A visual inspection was also performed to discard broken seeds, seeds correct but bad formation, and seeds with insect attack or damaged embryo regions. For germination, seeds were disinfected for 30 s in 70% ethanol and for 2 min in a solution of water and 10% commercial bleach (2–2.5% sodium hypochlorite; QBOA^®^, [Indústria Anhembi, Osasco, Brazil]), followed by three washes with sterile distilled water under a laminar flow hood. The seeds were then germinated on rolls of moistened germination paper in accordance with the Rules for Seed Analysis [18] in a seed germination chamber. The environment within the chamber consisted of a cycle of 30°C for 8 h in the light followed by 20°C for 16 h in the dark, for 5 days; a light-emitting diode (LED) lamp (6000 K, 5×15 W) was used for lighting.

### Growth parameter analyses

Primary root length (RL), seedling fresh matter (SFM), total root fresh matter (RFM), and total root dry matter (RDM) were determined from the 3^rd^ to 5^th^ day after sowing (DAS). For the RDM determination, roots were excised and dried in an oven at 60°C for 72 h. Three biological replicates were performed for each growth parameter, and each replicate consisted of three seedlings. *T-*test average comparisons were applied to group the means of these variables at a significance level of 5% using R software and the package *agricolae* [19,20]. The experiment was carried out using a completely randomized design.

The RL, SFM, RFM, RDM, and protein content values were used to determine the percentage of mid-parent heterosis (MPH) and best-parent heterosis (BPH) using the following formulas:

$$MPH=\frac{\mu F_{1}-\mu P}{\mu P}\times 100$$

$$BPH=\frac{\mu F_{1}-\mu_{best}P}{\mu_{best}P}\times 100$$

where *μF*_1_ is the mean of the F_1_ hybrids, *μP* is the mean of the two parental lines, and *μ*_*best*_*P* is the highest average among the best parental line.

### Proteomic analysis

For the proteomic analysis, the primary roots were sampled at the 5^th^ DAS. Protein extracts were prepared from three different biological samples (each sample contained 0.3 g RFM) using the 10% trichloroacetic acid (TCA)/acetone precipitation method described by Damerval et al. [21] with modifications. Briefly, the samples were ground under liquid nitrogen and suspended in 1 mL of chilled extraction buffer containing 10% (w/v) TCA (Sigma-Aldrich, St. Louis, USA) in acetone with 20 mM dithiothreitol (DTT; GE Healthcare, Little Chalfont, U.K.). The mixtures were maintained at -20°C for 1 h and then centrifuged at 16000 g for 30 min at 4°C. The resulting pellets were washed three times with cold acetone containing 20 mM DTT and subsequently air dried. The pellets were resuspended in buffer containing 7 M urea, 2 M thiourea, 1% DTT, 1 mM phenylmethylsulfonide, and 2% Triton X-100 (Sigma-Aldrich), vortexed for 30 min, and then centrifuged at 16000 g for 20 min at 4°C. The supernatants were then recovered, and their protein concentrations were determined using a 2-D Quant Kit (GE Healthcare).

### Protein digestion

Total protein samples (100 μg from each biological replication) were prepared in accordance with the methods of Reis et al. [22]. The samples were first precipitated using the methanol/chloroform method described by Nanjo et al. [23] and then resuspended in buffer containing 7 M urea and 2 M thiourea. Next, the samples were desalted on Amicon Ultra 3-kDa centrifugal filters (Merck Millipore, Darmstadt, Germany).

For digestion, 25 μL of 0.2% (v/v) RapiGest^®^ surfactant (Waters, Milford, USA) was added to each sample. The resultant mixtures were briefly vortexed and incubated in an Eppendorf Thermomixer^®^ (Eppendorf, Hamburg, Germany) at 80°C for 15 min. Next, 2.5 μL of 100 mM DTT was added, and the samples were incubated at 60°C for 30 min. Following the incubation, 2.5 μL of 300 mM iodoacetamide (GE Healthcare) was added to the samples, after which they were incubated in the dark for 30 min at 25°C. Then, 5 μL of 100 mM DTT was added to quench the excess iodoacetamide. Protein digestion was carried out by adding 20 μL of trypsin (50 ng μL^-1^) (Promega, Madison, USA) in 50 mM ammonium bicarbonate per sample, followed by incubation for 15 h at 37°C. Afterward, 10 μL of 5% (v/v) trifluoroacetic acid (TFA; Sigma-Aldrich) was added for RapiGest^®^ precipitation and trypsin activity inhibition, after which the samples were incubated at 37°C for 30 min followed by centrifugation at 16000 g for 20 min. The peptide mixtures were ultimately transferred to total recovery vials (Waters) for direct use in a Synapt G2-Si mass spectrometer (Waters).

### Nano-LC-ESI-MS/MS analyses

Nano-LC-electrospray ionization (ESI)-MS/MS analysis was carried out using a nanoAcquity UPLC (Waters) coupled to a Synapt G2-Si HDMS mass spectrometer (Waters). The peptide mixtures were separated by liquid chromatography by loading 2 μL of digested samples (1 μg of digested protein) onto a nanoAcquity UPLC 5 μm C18 trap column (180 μm×20 mm; Waters) and then onto a nanoAcquity HSS T3 1.8 μm analytical column (75 μm×150 mm; Waters) at a rate of 400 nL min^-1^ and temperature of 45°C. For peptide elution, the binary gradient consisted of MS water (Tedia, Fairfield, USA) and 0.1% formic acid (Sigma-Aldrich) as mobile phase A and of acetonitrile (Tedia) and 0.1% formic acid as mobile phase B. The gradient elution was started at 7% of solution B, increased to 40% of solution B for 91.12 min, increased to 99.9% of solution B until 92.72 min, persisted at 99.9% of solution B until 106 min, decreased to 7% of solution B until 106.1 min, and then persisted at 7% of solution B until the end of the run, at 120 min.

Mass spectrometry was performed both in positive and resolution (V) modes, with a full width at half maximum (FWHM) value of 35000 and ion mobility, and in data-independent acquisition (DIA) mode. The ion mobility separation (IMS) used an IMS wave velocity of 600 m s^-1^ (HDMS^E^); the transfer collision energy increased from 19 to 55 V in high-energy mode; the cone and capillary voltages were 30 and 2750 V, respectively; and the source temperature was 70°C. For time-of-flight (TOF) parameters, the scan time was set to 0.5 s in continuum mode, and the mass range was 50–2000 Da. The human [Glu1] fibrinopeptide B (Sigma-Aldrich) at 100 fmol μL^-1^ was used as an external calibrant, and lock mass acquisition was performed every 30 s. Mass spectrum acquisition was performed using MassLynx v.4.0 software (Waters).

### Protein identification and functional classification

Spectrum processing and database querying were performed using Progenesis QI for Proteomics v.2.0 software (Nonlinear Dynamics, Newcastle, UK) with the following parameters: Apex3D of 150 counts for the low-energy threshold, 50 counts for the elevated energy threshold, and 750 counts for the intensity threshold; a missed cleavage of one; a minimum fragment ion per peptide equal to two, a minimum fragment ion per protein equal to five, a minimum peptide per protein equal to two; fixed modifications of carbamidomethyl (C) and variable modifications of oxidation (M) and phosphoryl (STY) groups; a default maximum false discovery rate of 4%; a peptide score greater than four; and maximum mass errors of 10 ppm. Using the *Z*. *mays* protein sequence database, we queried candidate proteins against the UniprotKB database (www.uniprot.org). Label-free relative quantitative analyses were performed based on the ratio of protein ion counts. After the data were processed, only the proteins present in all three runs were considered. Based on the results of the ANOVA (*p*<0.05), differentially abundant proteins were considered up-regulated if their fold change (FC) was greater than 1.5 and down-regulated if their FC was less than 0.667. A Venn diagram of the identified proteins was constructed, and functional classification was performed using Blast2GO v.4.1 software (www.blast2go.com) [24]. The mass spectrometry proteomic data have been deposited in the ProteomeXchange Consortium [25] via the PRIDE [26] partner repository with the dataset identifier “PXD009436”.

To identify proteins with nonadditive characteristics, the total ion count (TIC) data were subjected to an analysis of variance [27] using the following linear model:

$$y_{ij}=\mu +T_{i}+e_{ij}\;(i:\;\in 1\ldots 3,\;and\;j:\;\in 1\ldots 3)$$

where *y*_*ij*_ is the *j* repetition of genotype *i*; *μ* is the general average, which is present in every *y*_*ij*_; *T*_*i*_ is the effect of treatment *i*; and *e*_*ij*_ is the random error. Based on the fit model, the contrast between the average hybrid and its parental lines was measured using a *t*-test assuming the following null hypothesis (H_0_):

$$\mu H=\frac{\mu P_{1}+\mu P_{2}}{2}$$

where *μH* is the hybrid mean and both *μP*_1_ and *μP*_2_ are the means of the parental lines [12].

The nonadditive proteins that were significantly more abundant in the hybrid than in the higher parental line were classified as “above-high parent abundance” (+ +). The proteins that significantly differed from those of the low parental line but not the high parental line were classified as “high parent abundance” (+), and the proteins that significantly differed from those of the higher parental line but not the lower parental line were classified as “low parent abundance” (-). The proteins that were significantly less abundant than those of the lower parental line were classified as “below-low parent abundance” (− −) [12].

## Results

### Heterotic effects on seedling growth

**Fig 1:**
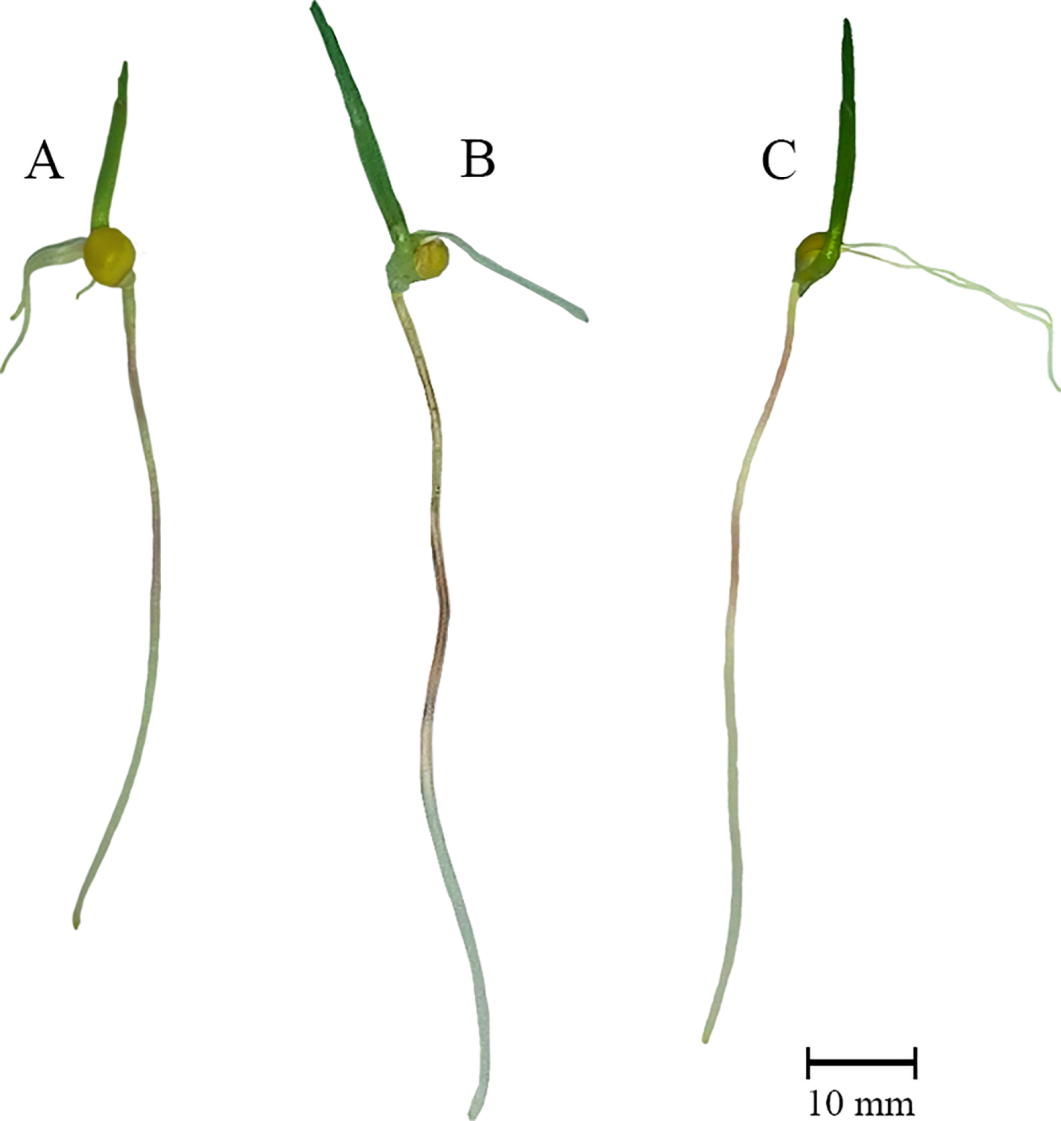
Popcorn seedlings at the 5^th^ DAS. A) Parental line L54 ♀; B) hybrid UENF/UEM01; C) parental line P8 ♂. Maize primary roots at the 5^th^ DAS display average lengths varying from 4.86±1.0 cm, 7.36±0.7 cm, and 6.76±1.2 cm for L54, hybrid UENF/UEM01, and P8, respectively. Nine observations were considered to account for each genotype.

The heterotic effects of the growth parameters of the seedlings were evaluated at the 5^th^ DAS (Fig 1 and Table 1). The MPH and BPH were calculated from the RL, SFM, RFM, RDM, and root protein content (Table 1). The heterotic effect analysis revealed positive effects for RL (27% and 8.2% for MPH and BPH, respectively) and RDM (19.5% and 2.4% for MPH and BPH, respectively). Negative effects were observed for RFM (-3.5% and -23.9% for MPH and BPH, respectively), SFM (-9.4% and -22.2% for MPH and BPH, respectively), and protein content (-11.8% and -7.2% for MPH and BPH, respectively) (Table 1).

**Table 1:** Growth parameter analysis and heterosis values from seedlings and primary roots of the popcorn hybrid UENF/UEM01 and both parental lines L54 ♀ and P8 ♂ at the 5^th^ DAS.

	Root Length (mm)	Root FM (mg)	Root DM (mg)	Seedling FM (mg)	Protein (mg g^-1^ RFM)
**L54**	48.6b*	36.1b	4.0a	229.0a	10.9a
**P8**	67.6a	62.8a	5.6a	313.7a	9.9a
**Hybrid**	73.6a	46.9ab	5.7a	253.4a	9.2a
**CV (%)**	12.9	17.0	15.6	23.4	22.3
**MPH (%)**	27.0	-3.5	19.5	-9.4	-11.8
**BPH (%)**	8.2	-23.9	2.4	-22.2	-7.2

*Means followed by the same letters in each column are not significantly different according to the *t-*test (*p*<0.05).

DM: dry matter; FM: fresh matter; RFM: root fresh matter; CV: coefficient of variation; MPH: mid-parent heterosis; BPH: best-parent heterosis. (n = 9)

In addition, we monitored the growth parameters of the seedling roots from the 3^rd^ to the 5^th^ DAS (S1 Fig). The RL between genotypes was significantly higher for the hybrid and parental line P8 ♂ than that for the parental line L54 ♀ at the 5^th^ DAS (S1 Fig). Compared with the parental lines L54 ♀ and P8 ♂, the hybrid exhibited significantly higher SFM at the 4^th^ DAS, but at the 5^th^ DAS, this difference was not observed (S1 Fig). Similar results were observed for RFM and RDM. Compared with the hybrid, the parental line L54 ♀ exhibited significantly lower RFM and RDM values at the 3^rd^ and 4^th^ DAS; however, these values did not significantly differ from those of the hybrid at the 5^th^ DAS (S1 Fig).

### Proteomic profiles

**Fig 2:**
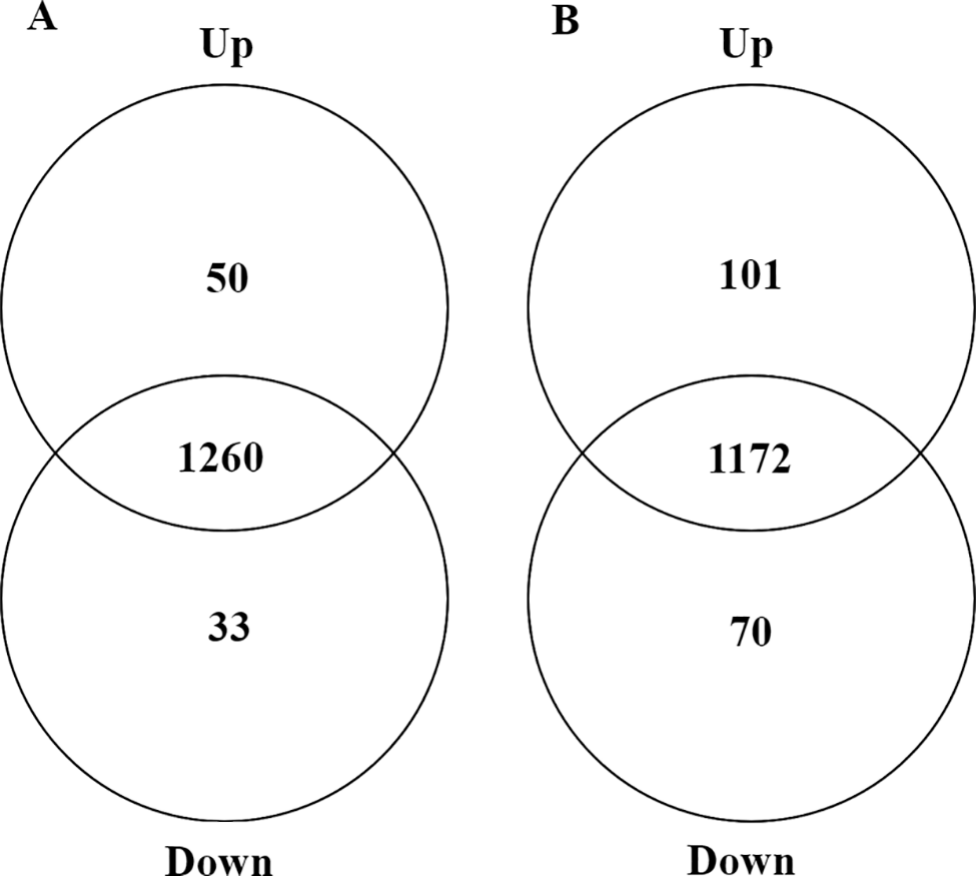
Venn diagram of identified proteins in the primary roots of popcorn plants; the data were generated from up- and down-regulated protein sets from comparisons between the hybrid UENF/UEM01 and its parental lines, L54 ♀ and P8 ♂. (A) Hybrid/L54 and (B) Hybrid/P8.

**Fig 3:**
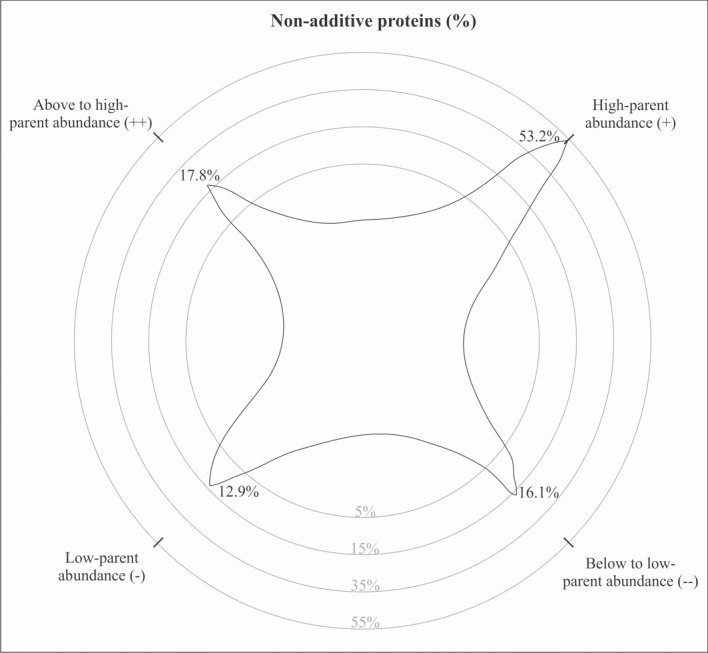
Percentage of nonadditive proteins in the hybrid UENF/UEM01 with respect to the mean of its parental lines, L54 ♀ and P8 ♂. (+ +) Above-high parent abundance; (+) high parent abundance; (-) low parent abundance; (− −) below-low parent abundance.

A total of 1343 proteins were identified in the primary roots of the popcorn hybrid UENF/UEM01 and parental lines L54 ♀ and P8 ♂ (S1 Table). Among these proteins, the FC data and ANOVA results (*p*<0.05) showed that the abundance of 1123 (83.6%) did not change, whereas 220 (16.4%) were differentially abundant between genotypes. Among the differentially abundant proteins, 50 and 33 proteins were up- and down-regulated when comparing the hybrid and parental line L54 ♀, respectively (Fig 2A), whereas 101 and 70 proteins were up- and down-regulated between the hybrid and parental line P8 ♂, respectively (Fig 2B). The abundance of 62 (4.6%) proteins significantly differed from the parental average (*t*-test, *p*<0.05), and based on such criteria, they were classified as nonadditively regulated proteins (Fig 3 and Table 2).

**Table 2:** Nonadditively regulated proteins identified in the primary roots of the popcorn hybrid UENF/UEM01 at the 5^th^ DAS.

Accession	Protein Description	Peptide Count	Confidence Score	Normalized Total Ion Count (TIC)			Fold Change Hybrid/Mean Parental Lines	T-test Value	Nonadditive Class*	Biological Process	Reference
				Mean L54	Mean P8	Mean Hybrid					
C4J5M2	AP-3 complex subunit delta	3	13.5	12722.8	11454.0	19641.4	1.62	0.030	(+)	Vesicle-mediated Transport	
B4FID1	40S ribosomal S17-4	2	19.9	9047.2	7494.7	15426.1	1.87	0.004	(+)	Translation	Marcon *et al*.2013
B6T1F1	60S ribosomal L19-3	4	43.9	1318.4	1689.8	2299.6	1.53	0.026	(+)	Translation	Marcon *et al*.2013
B6T379	40S ribosomal S15	2	20.0	23540.8	23639.4	38381.3	1.63	0.019	(+)	Translation	Guo *et al*.2013
B4FNW1	triosephosphate cytosolic	14	138.8	20680.5	14836.1	34631.0	1.95	0.041	(+)	Small Molecule Metabolic Process	López-Castillo *et al*.2016
Q06XS2	lipoxygenase chloroplastic-like	22	168.4	17824.1	27251.7	34955.5	1.55	0.037	(+)	Small Molecule Metabolic Process	Vale *et al*.2016
Q5K097	NADH dehydrogenase subunit 9 (mitochondrion)	5	27.5	5013.5	3531.0	6463.9	1.51	0.024	(+)	Small Molecule Metabolic Process	Vale *et al*.2016
Q9SWR9	dihydrolipoamide S-acetyltransferase	3	15.2	8471.0	5226.1	14563.1	2.13	0.030	(+)	Small Molecule Metabolic Process	Li *et al*.2007
Q9T0N7	phytase (plasmid)	15	124.3	24058.9	9977.1	32446.8	1.91	0.033	(+)	Small Molecule Metabolic Process	Hubel and Beck, 1996
Q9ZRQ5	phytase (plasmid)	16	122.9	43400.9	24064.4	55341.0	1.64	0.046	(+)	Small Molecule Metabolic Process	Hubel and Beck, 1996
B6SXF5	pathogenesis-related 1	8	80.4	124636.0	70752.5	154619.1	1.58	0.037	(+)	Signal Transduction	
C4J0W6	abscisic acid receptor PYR1-like	3	16.5	1261.2	534.4	1777.6	1.98	0.014	(+)	Signal Transduction	Leach *et al*.2011
A0A1D6IKX1	D-box ATP-dependent RNA helicase D 12-like	3	12.8	4636.9	4346.3	6856.7	1.53	0.008	(+)	RNA secondary structure unwinding	Meyer *et al*.2007
K7VW90	probable mediator of RNA polymerase II transcription subunit 37c	33	333.8	1345.1	1775.1	2974.4	1.91	0.011	(+)	RNA Editing	
B6T7B2	40S ribosomal S9	5	28.2	9017.6	7168.2	13143.0	1.62	0.006	(+)	Ribosome Biogenesis	Marcon *et al*.2013
K7V157	40S ribosomal S24	2	14.3	1197.2	1540.5	2522.5	1.84	0.015	(+)	Ribosome Biogenesis	Marcon *et al*.2013
O04014	40S ribosomal S6	6	40.9	43953.0	40880.6	67426.7	1.59	0.030	(+)	Ribosome Biogenesis	Marcon *et al*.2013
A0A1D6LFA7	heat shock 90-mitochondrial	14	76.3	1422.1	1643.7	2667.6	1.74	0.009	(+)	Protein Folding	Goff *et al*.2011
A0A1D6GQI0	NETWORKED 1A	6	33.6	6773.9	7562.3	11739.0	1.64	0.015	(+)	Protein Binding	
B4FN24	peroxiredoxin-2C	7	64.8	79705.9	67673.0	114615.6	1.56	0.010	(+)	Oxidation-reduction Process	Mohayeji *et al*.2014
A0A1D6KS86	NA	8	51.0	3023.8	2636.4	4306.4	1.52	0.001	(+)	NA	NA
B4F8K3	NA	3	16.6	2920.6	2542.8	4265.4	1.56	0.004	(+)	NA	NA
A0A1D6NUA0	Fanconi-associated nuclease 1 homolog isoform X1	3	12.9	683.8	885.4	1394.0	1.78	0.026	(+)	DNA Repair	
A0A1D6HHW2	ATP-dependent DNA helicase homolog chloroplastic isoform X1	4	17.2	3197.1	3000.9	5030.7	1.62	0.007	(+)	DNA Metabolic Process	Venu *et al*.2014
A0A1D6INH2	DNA polymerase zeta catalytic subunit	6	38.8	4653.9	4769.8	7290.8	1.55	0.005	(+)	DNA Metabolic Process	
Q8GS26	rp3	4	21.7	82039.7	64890.8	113744.2	1.55	0.002	(+)	Defense Response	Webb *et al*.2002
A0A1D6LQR2	isoleucine-tRNA cytoplasmic	6	28.6	12076.2	16459.6	23897.3	1.67	0.014	(+)	Cellular Amino Acid Metabolic Process	Meyer *et al*.2012
B6SWA0	phenylalanine ammonia-lyase	6	46.8	605.9	628.5	1173.5	1.90	0.006	(+)	Catabolic Process	Li *et al*.2007
A0A1D6EH08	probable O-methyltransferase 2	9	78.2	11768.5	15965.0	21872.4	1.58	0.040	(+)	Biosynthetic Process	Li *et al*.2007
B6SKV1	delta-1-pyrroline-5-carboxylate synthase	3	17.4	36471.1	22956.8	47371.6	1.59	0.029	(+)	Biosynthetic Process	
B6SZH6	malonyl-coenzyme A:anthocyanin 3-O-glucoside-6-O-malonyltransferase	3	14.6	885.3	1233.5	2458.3	2.32	0.002	(+)	Biosynthetic Process	
P41213	leucoanthocyanidin dioxygenase	9	64.4	22037.9	40825.1	52652.8	1.68	0.019	(+)	Biosynthetic Process	
Q93W19	bronze-2 partial	2	10.1	3151.6	3051.4	4682.3	1.51	0.025	(+)	Biosynthetic Process	Nash *et al*.1990
C0P4T5	aspartate-tRNA ligase cytoplasmic-like	11	62.9	169.5	192.2	318.4	1.76	0.001	(+ +)	tRNA Metabolic process	Meyer *et al*.2012
B4F9R4	60S ribosomal L8	9	52.9	1729.5	1785.7	3382.5	1.92	0.003	(+ +)	Translation	Marcon *et al*.2013
P45633	60S ribosomal L10-3	5	30.5	1333.4	1162.5	2355.0	1.89	0.000	(+ +)	Translation	Marcon *et al*.2013; Ferreyra *et al*.2013
B6U297	linoleate 9S-lipoxygenase 2-like	45	455.6	6365.7	9170.8	16918.3	2.18	0.002	(+ +)	Small Molecule Metabolic Process	
Q41739	thiazole biosynthetic enzyme, Thi4 family	10	70.0	12249.9	10679.6	18749.1	1.64	0.000	(+ +)	Small Molecule Metabolic Process	
B4FGG1	40S ribosomal S8	7	66.7	1542.6	1825.4	2833.2	1.68	0.005	(+ +)	Ribosome Biogenesis	Marcon *et al*.2013
Q08069	40S ribosomal S8	12	117.8	42391.5	43126.1	68446.1	1.60	0.005	(+ +)	Ribosome Biogenesis	Marcon *et al*.2013
A0A1D6DYX2	probable polyamine oxidase 2	2	9.5	3829.6	3834.2	7216.0	1.88	0.001	(+ +)	Polyamine Catabolic Process	Song *et al*.2016
A0A1D6MFZ1	GDSL esterase lipase At5g45920	3	16.4	4354.8	3884.7	6868.9	1.67	0.001	(+ +)	Lipid Metabolism	Chepyshko *et al*.2012
C0HIJ2	glutamine synthetase	3	22.7	3056.7	2955.1	4630.2	1.54	0.002	(+ +)	Biosynthetic Process	Li *et al*.2007; Huang *et al*.2011
P51108	NADPH-dependent reductase	6	36.3	4320.9	4366.1	8996.7	2.07	0.012	(+ +)	Biosynthetic Process	
A0A1D6I463	pentatricopeptide repeat-containing At5g61400	4	23.7	557.9	744.2	344.5	0.53	0.023	(-)	RNA Editing	Sosso *et al*.2012
K7VCN5	peroxidase 72-like	5	28.8	1678.1	3145.4	835.8	0.35	0.047	(-)	Oxidation-reduction Process	
B6TMW5	NA	6	35.9	9444.3	8593.2	3895.3	0.43	0.026	(-)	NA	NA
A0A1D6L870	PREDICTED: uncharacterized protein LOC103644200 isoform X1	7	31.4	5785.0	7512.7	3498.9	0.53	0.014	(-)	Embryo Development	
A0A1D6MIA5	replication factor A 1-like	4	17.0	828.4	351.3	95.8	0.16	0.045	(-)	DNA Metabolic Process	
A0A1D6KCZ2	alanine aminotransferase	11	68.0	1490.5	1943.5	1113.6	0.65	0.040	(-)	Catabolic Process	Han *et al*.2016
A0A1D6NMK4	probable zinc protease	3	12.9	3281.9	4151.8	2128.0	0.57	0.005	(-)	Catabolic Process	
K7TSA0	26S proteasome non-ATPase regulatory subunit 1 homolog A-like	5	22.9	8806.6	11785.6	6718.3	0.65	0.043	(-)	Catabolic Process	Vale *et al*.2016; Yao *et al*.2005; Mohayeji *et al*.2014
B4FP25	40S ribosomal S19	6	55.0	53423.4	51331.5	27996.3	0.53	0.005	(− −)	Translation	Marcon *et al*.2013
A0A1D6L3F0	serine threonine-kinase SMG1-like	2	8.3	97.6	104.0	43.1	0.43	0.005	(− −)	Small Molecule Metabolic Process	
B4FG53	malate chloroplastic	8	55.5	1288.8	1217.3	569.2	0.45	0.000	(− −)	Small Molecule Metabolic Process	Vale *et al*.2016
K7U473	pentatricopeptide repeat-containing At4g02750-like	2	8.5	1044.5	1015.0	578.9	0.56	0.009	(− −)	RNA Editing	Sosso *et al*.2012
B4FHX3	60S ribosomal L23	5	42.8	26790.7	21437.0	12193.6	0.51	0.049	(− −)	Ribosome Biogenesis	Marcon *et al*.2013
A0A1D6HCC1	intracellular transport USO1	13	66.7	534.6	441.7	166.7	0.34	0.004	(− −)	Protein Translocation	
A0A1D6KE00	importin subunit beta-1	14	86.3	5231.7	5094.1	3256.3	0.63	0.000	(− −)	Protein Translocation	
B4FT54	DnaJ subfamily B member 5	2	10.2	754.7	709.3	174.6	0.24	0.000	(− −)	Protein Folding	
B4FBI6	fumarylacetoacetase	2	9.5	544.1	622.2	276.2	0.47	0.003	(− −)	Catabolic Process	Yu *et al*.2014
K7U025	haloacid dehalogenase-like hydrolase domain-containing 1A	6	30.7	6532.4	6139.1	3896.7	0.62	0.005	(− −)	Carbohydrate Metabolic Process	Caparrós-Martín *et al*.2013

* Nonadditive classes: above-high parent abundance (+ +), below-low parent abundance (− −), high parent abundance (+), and low parent abundance (−).

### Nonadditively regulated proteins

Among the 62 nonadditively regulated proteins, 33 (53.2%) were classified as high parent abundance (+), eight (12.9%) as low parent abundance (-), 11 (17.8%) as above-high parent abundance (+ +), and 10 (16.1%) as below-low parent abundance (− −) (Fig 3). The results demonstrated that the high parent abundance class constituted the most frequent heterotic effect on the primary roots of the UENF/UEM01 hybrid (Fig 3).

### Functional classification

**Fig 4:**
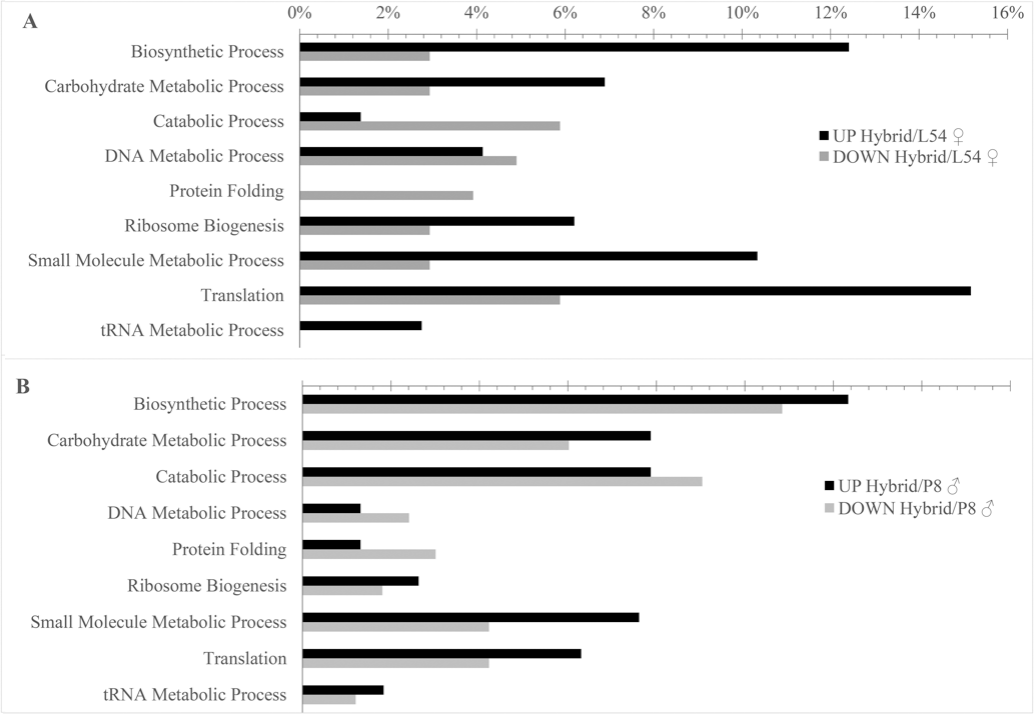
**Biological process classification of differentially abundant proteins identified in the primary roots of the popcorn hybrid UENF/UEM01 in relation to its parental lines, L54 ♀ (A) and P8 ♂ (B)**.

The differentially abundant proteins were classified into nine biological processes. Among these processes, six (biosynthetic process, carbohydrate metabolic process, small molecule metabolic process, ribosome biogenesis, translation, and tRNA metabolic process) were up-regulated more in the hybrid than in the parental lines L54 ♀ and P8 ♂, whereas three (catabolic process, DNA metabolic process, and protein folding) were downregulated (Fig 4). Nonadditively regulated proteins were associated with 22 biological processes (Table 2), of which small molecule metabolic processes, biosynthetic processes, translation, and ribosome biogenesis were the most abundant, representing altogether 45.2% of the total nonadditive proteins.

Finally, all the identified and classified proteins were classified into 10 biological processes; organic acid metabolic processes were the most represented, making up 36% of proteins (S2 Fig).

## Discussion

UENF/UEM01 is a high-technology hybrid with the best production and quality stability among different environmental conditions observed in Brazil [28]. Its parental lines L54 and P8 generate hybrids exhibiting some of the best attributes for grain yield within the active germplasm bank and good grain expansion capacity [29]. Other important features of the UENF/UEM01 parental lines are the tolerance to *Fusarium* spp. and *Puccinia polysora* of the L54 line [30,31] and the resistance to *Bipolaris maydis* and *Exserohilum turcicum* of the P8 line [32].

Heterosis is widely studied because of its quantitative traits of economic importance and its dependence on the expression of many genes beginning at the onset of plant development. Root development is affected by hybrid vigor and is an important model for studying hybrid vigor in juvenile tissues [14]. In our study, primary RL and RDM were the most consistent heterotic traits for popcorn; both displayed positive and high MPH values (27 and 19.5%, respectively) (Table 1).

Others studies examining hybrids of crosses between dent corn and flint corn have reported positive values of MPH for primary RL, longitudinal size of cortex cells, and lateral root density, providing a larger root absorption surface area and growth rates beginning at early stages of plant development [33]. Thus, the genetic combination of hybrids causes small differences in various structures that together can lead to more efficient or alternative processes that allow the genotype to exhibit hybrid vigor.

Transcriptional and translational expression profiles have revealed less overlap of nonadditively expressed genes and significant differences in the degree of heterosis between different maize hybrid tissues, e.g., apical shoots and primary roots [16,34]. These observations suggest the presence of tissue-specific expression of additive and nonadditive effects in hybrids and the nonadditive expression of many genes in primary roots [34,35].

In our study, most of the nonadditively regulated proteins (66.2%) identified in the primary roots of the UENF/UEM01 popcorn hybrid displayed a prevalence of high (dominant effect) and above-high parent (overdominant effect) abundance (Fig 3).

Nonadditive variance is a phenomenon observed in hybrid phenotypes for several variables (mainly quantitative traits), evidencing the effect of hybrid vigor [36]. As result of nonadditive variance, the mean of the hybrid of a specific trait cannot be simply predicted from the performance of both parents, and this phenomenon is called heterosis [37].

Proteins with nonadditive regulation in hybrids are candidates as biomarkers for heterosis [38]. Therefore, we have discussed the main metabolic processes associated with such nonadditive proteins with high (dominant effect) and above-high parent (overdominant effect) abundance in the hybrid compared with those in the parental lines (Table 2).

### Small molecule metabolic process-related proteins

In this study, 16% of the nonadditively regulated proteins were associated with small-molecule metabolic processes, and increasing trends among these proteins were observed. Proteins associated with fatty acid metabolism, energy production, and phosphate availability were identified. Two lipoxygenase (LOX) enzymes (Q06XS2 and B6U297) were identified as exhibiting a high-parent abundance in the primary roots of the UENF/UEM01 hybrid (Table 2). These proteins are associated with fatty acid metabolism and catalyze the formation of hydroperoxyl derivatives by oxygenation of polyunsaturated fatty acids [39]. Such derivatives can act as signaling molecules in several developmental processes or can be directed as acyl derivatives to the Krebs cycle for energy production [12,39]. Thus, a higher abundance of these proteins may promote increased energy production and molecular signaling during primary root development. However, lower abundances of LOX1 were detected in the ear shoots of flint corn hybrids that exhibited positive heterosis for ear size and kernel number [40] as well as in the roots of papaya hybrids that exhibited positive heterosis for root size [12]. These observations may indicate differential organ- and species-specific abundances of lipoxygenases in hybrids that exhibit positive heterosis for different traits.

Three proteins that show high parent abundance and are directly involved with glucose metabolism and the respiratory chain were identified in the UENF/UEM01 hybrid (Table 2): a cytosolic triosephosphate enzyme (B4FNW1) involved in gluconeogenesis and the glycolysis pathway [41]; a dihydrolipoamide S-acetyltransferase (Q9SWR9) subunit of the maize mitochondrial pyruvate dehydrogenase complex (PDC) [42]; and a NADH dehydrogenase subunit nine structural protein (Q5K097) of the mitochondrial NADH-oxidizing protein complex of the respiratory chain [43]. Thus, a higher abundance of these proteins could be related to increased glucose metabolism and energy production in the primary roots of UENF/UEM01 hybrids. However, a low abundance of proteins belonging to both the PDC and NADH dehydrogenase complex was detected in the roots of papaya hybrids [12], suggesting in this case that energy consumption/production was lower in the hybrids than that in the parental lines.

Phytase enzymes (Q9T0N7, Q9ZRQ5) are types of phosphohydrolases that can be differentiated from nonspecific acid phosphatases by their ability to initiate the dephosphorylation of phytate (inositol hexakisphosphate). Phytate is the major storage form of phosphate in seeds, and in maize plants, phytase activity is consistently observed in the pericycle, root endodermis [44], and shoots [45], as these organs are involved in the use of soil-borne phytate. Thus, higher contents of phytate may contribute to RDM, and a higher abundance of phytase in the primary roots of the UENF/UEM01 hybrid may result in enhanced phytate dephosphorylation and phosphorus availability during the early stages of root development.

Finally, a thiazole biosynthetic Thi4 family enzyme (Q41739) exhibiting high parent abundance in the UENF/UEM01 hybrid was detected. This protein is involved in thiazole biosynthesis and catalyzes the conversion of NAD and glycine to adenosine diphosphate (ADT); Q41739 also functions in hybrid adaptations to various stress conditions and in DNA damage tolerance [46]. Moreover, many studies have reported that thiamine biosynthesis is up-regulated during plant adaptation responses to abiotic stress, including salt, cold, flooding, heat, drought, and osmotic stress [46].

### Biosynthetic process-related proteins

Seven nonadditive proteins, approximately 13% of which exhibited high or above-high parent abundance, were associated with biosynthetic processes (Table 2). Six proteins were identified as enzymes acting in several steps of the anthocyanin biosynthesis pathway, whereas two were related to the biosynthesis of the amino acids glutamine and proline (Table 2).

The proteins belonging to the anthocyanin biosynthesis pathway include an NADPH-dependent reductase (P51108), in this case a bifunctional enzyme showing both dihydroflavonol 4-reductase and dihydrokaempferol 4-reductase activity [47]; a leucoanthocyanidin dioxygenase (P41213), which oxidizes leucoanthocyanidins into anthocyanidins [48]; a malonyl-coenzyme A:anthocyanin 3-O-glucoside-6-O-malonyltransferase (B6SZH6), which is involved in the addition of a malonyl group to a cyanidin derivative during anthocyanin biosynthesis [49]; a bronze-2 protein (Q93W19) that is a glutathione S-transferase enzyme and acts during the later stages of the anthocyanin pathway, resulting in the deposition of red and purple pigments in the vacuoles of plant maize tissues [50]; and, finally, a probable *O*-methyltransferase 2 (A0A1D6EH08) that methylates a variety of secondary metabolites, including phenylpropanoids, flavonoids, and alkaloids [51].

In this context, anthocyanin levels are significantly higher in hybrids than those in inbred lines, resulting from the above-high parent expression of the seedling *Pl* locus [9]. The *Pl* gene product is a transcription factor that epistatically regulates the expression of several genes that control anthocyanin production [9], resulting in the nonadditive accumulation of gene products in a similar manner to that observed in our study.

The proteins that were identified as involved in amino acid biosynthesis included a glutamine synthetase (C0HIJ2) and a delta-1-pyrroline-5-carboxylate synthase (B6SKV1); both proteins exhibited high-parent abundances in the UENF/UEM01 hybrid (Table 2). Glutamine synthetase is a metabolism-related enzyme that is involved in the biosynthesis of glutamine from glutamate [52], whereas delta-1-pyrroline-5-carboxylate synthetase is involved in both the glutamyl kinase and glutamic-semialdehyde dehydrogenase activities during the first two steps of proline biosynthesis [53]. These amino acids may accumulate in the hybrids due to a demand for resynthesized proteins or for molecular precursors with regulatory or metabolic functions [54,55].

### Ribosome biogenesis, tRNA, and translation-related proteins

In this study, we identified several ribosomal and translation-related proteins that exhibited a nonadditive abundance in the popcorn hybrids. These proteins act as structural components of short and large ribosomal subunits, including the 40S ribosomal S6 (O04014), S8 (B4FGG1, Q08069), S9 (B6T7B2), S15 (B6T379), and S17-4 (B4FID1), and S24 (K7V157) subunits, as well as the 60S ribosomal L8 (B4F9R4), L10-3 (P45633), and L19-3 (B6T1F1) subunits (Table 2). tRNA-related proteins can be co- or posttranscriptionally modified by a larger collection of chemical moieties, including same proteins that were identified in our work, such as aspartate-tRNA ligase cytoplasmic-like (C0P4T5) and isoleucine-tRNA cytoplasmic (A0A1D6LQR2) (Table 2). These proteins participate mainly in the tRNA anticodon loop [56] and are directly related to cytoplasmic protein synthesis in mitochondria and the same tRNA [57] and binding protein in ribosomes [58].

Proteomic studies on the roots of semiflint and dent corn hybrids have demonstrated that the protein metabolism functional category represents the most abundant class of nonadditive proteins; 60% are represented by ribosomal proteins exhibiting high or above-high parent abundance [14].

The increased abundance of ribosomal proteins may be related to increased rates of protein synthesis during the early manifestation of heterosis, as suggested by [14]. Protein synthesis plays an important role in controlling cell growth and development and is positively correlated with the transcript accumulation of genes encoding ribosomal proteins. However, in the roots of papaya hybrids, most ribosomal proteins are classified as below-low parent abundance [12], suggesting that heterosis differs depending on species, tissue type, and stage of development [8].

## Conclusions

The present study provides a quantitative comparison between the proteomic profiles of young primary roots and growth parameters of the UENF/UEM01 popcorn hybrid and its parental lines L54 ♀ and P8 ♂. Our results suggest that positive heterosis for primary RL and seedling DM of the hybrids is associated with changes in the abundance of proteins of primary roots related to the optimization of energy metabolic processes, protein translation, and ribosome biogenesis; these changes allow the hybrid to develop faster during the early stages in conjunction with lower amounts of protein and water (all fresh matter measured and concentrations of proteins), indicating an optimization of resources available for development. Thus, our results contribute to a better understanding of the molecular events that regulate heterosis in popcorn at an early stage in plant development.

## Supporting information

S1 Table Complete list of identified proteins for the hybrid UENF/UEM01 and its parental lines L54 ♀ and P8 ♂. (XLSX)

S1 Fig Growth parameters of root length (RL) (A), seedling fresh matter (SFM) (B), root fresh matter (RFM) (C), and root dry matter (RDM) (D) for the popcorn hybrid UENF/UEM01 and its parental lines L54 ♀ and P8 ♂ from the 3^rd^ to 5^th^ DAS. Means followed by the same letters within the same DAS are not significantly different according to the *t*-test (*p*<0.05).
(TIF)

S2 Fig Biological processes of total proteins identified for hybrid UENF/UEM01 and its parental lines L54 ♀, and P8 ♂. (TIF)
